# Detection and Processing Techniques of FECG Signal for Fetal Monitoring

**DOI:** 10.1007/s12575-009-9006-z

**Published:** 2009-03-27

**Authors:** MA Hasan, MBI Reaz, MI Ibrahimy, MS Hussain, J Uddin

**Affiliations:** 1 Department of Electrical and Computer Engineering, International Islamic University Malaysia, Gombak, 53100, Kuala Lumpur, Malaysia; 2 Department of Electrical, Electronic and Systems Engineering, Universiti Kebangsaan Malaysia, 43600 UKM, Bangi, Selangor, Malaysia

**Keywords:** ECG, MECG, FECG, FHR, QRS complex, FPGA

## Abstract

Fetal electrocardiogram (FECG) signal contains potentially precise information that could assist clinicians in making more appropriate and timely decisions during labor. The ultimate reason for the interest in FECG signal analysis is in clinical diagnosis and biomedical applications. The extraction and detection of the FECG signal from composite abdominal signals with powerful and advance methodologies are becoming very important requirements in fetal monitoring. The purpose of this review paper is to illustrate the various methodologies and developed algorithms on FECG signal detection and analysis to provide efficient and effective ways of understanding the FECG signal and its nature for fetal monitoring. A comparative study has been carried out to show the performance and accuracy of various methods of FECG signal analysis for fetal monitoring. Finally, this paper further focused some of the hardware implementations using electrical signals for monitoring the fetal heart rate. This paper opens up a passage for researchers, physicians, and end users to advocate an excellent understanding of FECG signal and its analysis procedures for fetal heart rate monitoring system.

## 1. Introduction

Fetal heart rate (FHR) monitoring is a routine for obtaining significant information about the fetal condition during pregnancy and labor. The characteristics of the fetal electrocardiogram (FECG), such as heart rate, waveform, and dynamic behavior, are convenient in determining the fetal life, fetal development, fetal maturity, and existence of fetal distress or congenital heart disease. The FHR may change as the fetus responds to conditions in the uterus. An abnormal FHR or pattern may mean that the fetus is not getting enough oxygen or there are other problems. Sometimes an abnormal pattern also may mean that an emergency or cesarean delivery is needed. During pregnancy, the motivation for monitoring the fetal is to recognize pathologic conditions, typically asphyxia, with sufficient warning to enable intervention by the clinician. Therefore, FHR carries a significant importance of clinical perspectives.

### 1.1. Clinical Significance of Fetal Heart Rate

During the last decades, FHR monitoring has been extensively used for intrapartum and antepartum monitoring to assess fetal well-being. It is commonly used as a screening modulus of the fetus to detect in advance possible fetal problems that could result in irreversible neurological damage or even fetal death during labor. FHR has become a routine physiological measurement both during labor and during the antenatal period when certain pregnancy risk factors have been identified. The normal pattern of FHR is well established and easily recognized while other patterns (e.g., decelerations, loss of high-frequency variability, and pseudo-sinusoidal) can be indicative of fetal asphyxia. Although detection of fetal compromise is one benefit of fetal monitoring, there are also risks, including false-positive tests that may result in unnecessary surgical intervention. Therefore, it has been classified that there are two methods of FHR monitoring during labor and delivery.

Auscultation is a method of listening to the fetal heartbeat from mother's abdomen. Electronic fetal monitoring is a procedure in which instruments are used to record the heartbeat of the fetus and the contractions of the mother's uterus during labor. Sometimes, auscultation and electronic fetal monitoring are used together to determine the status of the fetus perfectly. Either method is a good way to measure how well the baby is doing during labor and delivery. The choice of which method is used depends on how a pregnant women's labor is going and her risk of problems.

#### 1.1.1. Auscultation

Auscultation involves listening to one's baby's heartbeat. There are two ways of listening to the fetal heartbeat with auscultation:

1. A *Doppler* ultrasound device is a small device that is pressed against mother's abdomen. This device uses a form of ultrasound to convert sound waves into signals of fetal heartbeat.

2. A special device like a stethoscope called a fetoscope is placed in the ears of doctor or nurse. The open end is pressed on mother's abdomen. The fetoscope allows fetal heartbeat to be heard clearly. It is used less often than Doppler.

The heart rate of the fetus will be monitored before, during, and just after a contraction or nonstop during labor to tell how the fetus reacts to the contraction. Electronic monitoring can be the better option for fetal monitoring if abnormal patterns are found in auscultation method.

#### 1.1.2. Electronic Fetal Monitoring

Electronic fetal monitoring uses special equipment to measure the response of the FHR to contractions of the uterus. It provides an ongoing record that can be read by the doctor or nurse. Electronic fetal monitoring can be external (outside), internal (inside), or both. The pregnant woman needs to stay in bed during both types of electronic monitoring, but she can move around and find a comfortable position.

##### Internal monitoring

Internal monitoring involves placement of a small plastic device about the size of a pencil eraser through the cervix. A spiral wire called the fetal scalp electrode is placed just beneath the skin of the fetal scalp. The fetal scalp electrode then transmits direct information about the fetal heart rate through a wire to the fetal monitor that prints out this information. Because the internal fetal monitor is attached directly to the baby, the FHR signal is sometimes much clearer and more consistent than with an external monitoring device. However, there may be a slight risk of infection with internal monitoring. The scalp electrode may also cause a mark or small cut on the baby's head, but this usually heals quickly [1].

##### External monitoring

By definition, external fetal monitoring is done through the skin and is not meant to be invasive. Sensitive electrodes are placed on mother's abdomen over conducting jelly that can sense both FHR and the strength and duration of uterine contractions. The nonstress test (NST) is another way of externally monitoring the baby. The NST can be done as early as the 27th week of pregnancy and it measures the FHR accelerations with normal movement [2]. The contraction-stress test is a final method of externally monitoring the baby. This test measures the ability of the placenta to provide enough oxygen to the fetus while under pressure during the contractions [2]. In fact, there are no known risks by using the fetoscope, Doppler, or external monitoring for FHR. Some FHR monitoring techniques are highlighted in terms of patient's benefits and maintenance.

### 1.2. FHR Monitoring Techniques

Fetal heart rate analysis has become a widely accepted means of monitoring fetal status [3]. The most familiar means of acquiring the FHR is Doppler ultrasound. In addition, the FHR monitoring is also done by considering fetal magnetocardiogram (FMCG) that uses superconducting quantum interference device magnetometers [4]. Apart from this, fetal phonocardiography (FPCG) allows the heart sounds to be detected for FHR monitoring [5]. The majority of FHR analysis technique is performed using a bedside monitor over a relatively short period, with the mother-to-be in a recumbent position. All of the above techniques that are mentioned have been successfully used for FHR monitoring, although the initial choice was which of the above techniques would be employed. Obviously, a fetal scalp electrode cannot be used antepartum period as there is a great risk to cause a mark or small cut on the fetal head; the instrumentation required for the acquisition of the FMCG is too cumbersome for ambulatory use; while fetal phonocardiography was felt to be too susceptible to movement artifacts effects. Therefore, the Doppler ultrasound and the abdominal FECG (as it is commonly referred to) are the most viable options for the monitoring of FHR.

Currently, Doppler ultrasound and FECG have proven to be reliable techniques for monitoring FHR. The FHR monitoring using the Doppler ultrasound is widely used and appropriate because an invasive test cannot be used daily [6]. The advantage of the Doppler ultrasound technique is that it can be virtually assured that a recording of FHR will be obtained. The disadvantages of such systems require intermittent repositioning of the transducer and they are only suitable for use with highly trained midwifes. The ultrasound transducer is problematic and uncomfortable while the procedure involves launching a 2-MHz signal towards the fetus [7]. The use of Doppler ultrasound (noninvasive manner) is not suitable for long periods of FHR monitoring [8]. This may involve skillful placement and continual repositioning of the transducer, which would be a severe problem for long-term ambulatory use. It may cause records of uncertain accelerations or decelerations and true abrupt changes can be misinterpreted as noise [9-11]. The major limitation of the Doppler ultrasound technique is its sensitivity to movement. The movement of the mother can result in Doppler-shifted reflected waves, which are stronger than the cardiac signal. This Doppler ultrasound technique is inappropriate for long-term monitoring of the FHR, as it requires the patients to be bed-rested [12]. Moreover, the detection of the heartbeat using Doppler ultrasound relies upon a secondary effect (the mechanical movement of the heart) and is therefore not as accurate for beat-to-beat analysis as detection of the QRS complex. Allied to this drawback is the fact that most Doppler systems rely upon some form of averaging to produce their FHR data.

In contrast, methods utilizing the abdominal electrocardiogram (AECG) have a greater prospect for long-term monitoring of FHR and fetal well-being using signal-processing techniques [13]. The AECG signal can also be used for antepartum noninvasive FHR determination through the detection of small fetal cardiac potentials at the surface of the maternal abdomen [14]. The AECG can be used to produce true R–R interval data, which is suitable for heart rate variability studies if required. Its advantage is that it is completely noninvasive and unobtrusive, has comparatively low power requirements, and can be used over extended (e.g., 24 h) periods. The method additionally allows the maternal heart rate (MHR) to be recorded since the MECG is also detected from the AECG. It is advantageous of using AECG to extract FECG with the additional information compared to using Doppler ultrasound [15]. Some new highly accurate techniques are reported for monitoring the FHR [16,17]. The major disadvantage with this technique is that the acquisition of the FECG cannot be guaranteed and often has a very low signal-to-noise ratio (SNR) because of the interference caused by MECG, electromyogram (EMG), and motion artifact in determining the FHR from the AECG signal. To overcome the above problems, some multiple-lead algorithms use the thoracic MECG to cancel the abdominal MECG [18], though this is inconvenient for the patient during long-term monitoring. Hence, to make the AECG suitable for the detection of the FECG, the SNR must be enhanced. The decision was therefore made to base the investigation on the possibility of constructing an ambulatory FHR recorder around the acquisition of the abdominal FECG.

The FECG is an electrical signal that can be obtained noninvasively by applying a pair of electrodes to the abdomen of a pregnant woman [14]. Therefore, detection of FECG signals with powerful and advance methodologies is becoming a very important requirement in biomedical engineering for the interest in FECG signal analysis in clinical diagnosis and biomedical applications. The FECG contains potentially valuable information that could assist clinicians in making more appropriate and timely decisions during labor, but the FECG signal is vulnerable to noise and difficulty of processing it accurately without significant distortion has impeded its use [19-22]. A number of difficulties and complication are associated with recording the AECG. The signal-processing algorithm needs to remove the MECG complexes, reduce the effects of motion artifact, muscle noise, and power line interface, and then enhance the fetal QRS complexes before they can be consistently detected. Therefore, to get proper information of the FHR and fetal status, it is necessary to improve the SNR of the abdominal signal.

Although there are still limitations for monitoring the FHR perfectly, currently, there is a significant amount of effort being done to improve SNR of FECG signal. Traditional system reconstruction algorithms have various limitations and considerable computational complexity and many show high variance. Up-to-date advances in technologies of signal-processing and mathematical models have made it matter-of-fact to develop advanced FECG detection, extraction, and analysis techniques. Ranges of mathematical techniques and artificial intelligence (AI) have acknowledged comprehensive attraction. Mathematical models incorporate wavelet transform (WT), time–frequency approaches, Fourier transform, statistical signal analysis, and higher-order statistics. AI approaches towards signal recognition include artificial neural networks (ANN) [23], self-organizing map (SOM) neural network [24], finite impulse response (FIR) neural network [25], and fuzzy logic system [26]; a technique combining the adaptive noise canceller and adaptive signal enhancer [27] in a single recurrent neural network is being used for the processing of AECG signal. Singular value decomposition [13] and IIR adaptive filtering combined with genetic algorithms [28] are also used for FHR monitoring. The computerized analysis of FHR monitoring based on the combination of wavelet analysis and artificial neural network is a very promising technique in objective intrapartum diagnosis of fetal hypoxia. In the field of FECG and FHR extraction, various research efforts have been carried out, including subtraction of an averaged pattern, matched filtering, adaptive filtering, orthogonal basis functions, fractals, temporal structure, frequency tracking, polynomial networks, wavelets, and real-time signal processing.

Methods of extracting FECG from the AECG have been recently introduced for the monitoring of FHR. These methods can be classified with respect to the principle ideas of signal processing as follows: threshold technique, spectral analysis, linear combinations, or weighted sums. The extraction of FECG from the complex signal (mother and fetus) can be reframed in a more efficient manner using blind source separation (BSS) methods such as principal component analysis [29] and independent component analysis (ICA) [16,29]. Wavelet transform is well fitting to nonstationary signals like ECG. The combination of wavelet analysis and BSS methods also shows potential attitude for the separation of the maternal and fetal signals from ECGs. Blind-adaptive-filtering [30] tactic overcomes the theoretical limitations in applying conventional BSS methods based on ICA for FECG signal extraction problem. So far, research and extensive works have been made in the area, developing better algorithms, upgrading existing methodologies, and improving detection techniques to reduce noise and acquire accurate FECG signals to obtain reliable information about the fetal state thus assuring fetal well-being during pregnancy period.

The aim of this paper is to explore by highlighting the different approach and methodologies for monitoring the FHR. Therefore, this paper firstly gives a brief clarification about FECG signal and a short historical background of FECG signal analysis as well. This is followed by highlighting the up-to-date detection, extraction processing, and classification methods of FECG signal along with a comparison study. Lastly, some hardware implementations of FECG signal analysis to monitor FHR have been discussed.

## 2. Anatomical and Physiological Background of FECG

### 2.1. Clinical Importance of FECG Morphology

**Figure 1:**
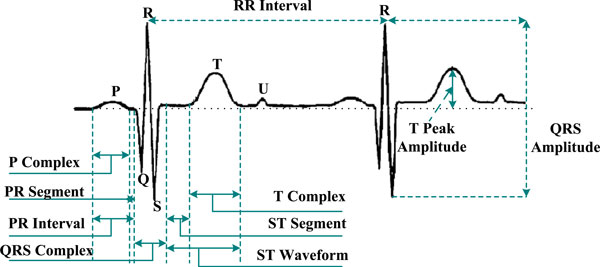
**FECG is showing key features: the PQRST complex**.

Biomedical signal means a collective electrical signal acquired from any organ that represents a physical variable of interest where the signal is considered in general a function of time and is describable in terms of its amplitude, frequency, and phase. FECG is a biomedical signal that gives electrical representation of FHR to obtain the vital information about the condition of the fetus during pregnancy and labor from the recordings on the mother's body surface. The FECG signal is a comparatively weak signal (less than 20% of the mother ECG) and often embedded in noise. The FHR lies in the range from 1.3 to 3.5 Hz and sometimes it is possible for the mother and some of the FECG signals to be closely overlapping. The FHR monitoring enables accurate measurement of fetal cardiac performance including transient or permanent abnormalities of rhythm [31]. Sometimes, the FECG is the only information source in early-stage diagnostic of fetal health and status. The FECG is very much related to the adult ECG, containing the same basic waveforms including the P wave, the QRS complex, and the T wave. The PQRST complex as shown in Figure 1 is an electric signal produced by the contraction of the heart's muscle called *myocardium*. It is composed of three parts:

■ The P wave occurs at the beginning of atrial contraction.

■ The QRS complex is associated with the contraction of the ventricles. Due to the magnitude of the R wave, it is extremely reliable.

■ The T wave corresponds to the repolarization phase, which follows each heart contraction.

The R–R interval leads to the heartbeat frequency that gives useful information for the heart condition.

Morphologies of interest include the shape, size, and duration of individual and groups of FECG waveforms as well as the various ratios relating these quantities to each other. The fetal signal achieved from the maternal abdomen typically has low amplitude and an unfavorable signal-to-noise ratio from which the FHR can hardly be detected [32]. The signal-processing algorithm needs to eliminate the MECG complexes, reduce the effects of motion artifact and muscle noise, and then enhance the fetal QRS complexes before they can be consistently detected.

### 2.2. Fetal ECG Signal Enhancement

The human heart generates the quintessential biological signal: the heartbeat. A recording of the cardiac-induced skin potentials at the body's surface, an electrocardiogram, reveals information about atrial and ventricular electrical activity. Abnormalities in the temporal durations of the segments between detections or of the intervals between waves in the ECG, as well as their relative heights, serve to expose and distinguish cardiac dysfunction. The most commonly used technique to improve the SNR for repetitive signals, such as the FECG, is averaging [33,34]. A major flaw in signal averaging is that it tends to remove short-term changes in the ECG waveform. Further, a single ECG complex of poor quality may have an undue influence or indeed distort the resulting ECG average. To avoid this, ECG complexes that satisfy an appropriate quality criterion are included in the averaging process. However, this may distort the time of occurrence information and makes it difficult to correlate changes in the ECG waveform to those in the cardiotocogram (CTG) or other events in labor which is important for a proper assessment of the fetal condition. The CTG is a technical means of recording the fetal heartbeat and the uterine contractions during pregnancy. Mainly, these recordings are done by two separate transducers, one for the measurement of the fetal heart rate and a second one for the uterine contractions. A typical CTG reading is printed on paper and/or stored on a computer for later reference of physician. CTG is being used to identify signs of fetal distress. Further, the existences of significantly large low-frequency noise components, which are correlated, such as baseline shifts, serve to reduce the effectiveness of averaging. The optimum solution to the problem of FECG enhancement for feature extraction would require the removal of the baseline shifts as well as matching the digital filter spectrum to that of the FECG. This way, the distortion of the features of the ECG is kept to a minimum by the signal processing. Therefore, it is needed to enhance FECG signal-processing technique to assess the status of the fetus by monitoring the FHR.

### 2.3. A Brief History of Fetal Electrocardiography

Acoustic FHR monitoring captures the beating activity of the fetal heart valves opening and closing using an acoustic sensor placed on the mother's abdomen [35]. As long ago as 1818, a physician detected fetal heartbeats by listening to a fetus from the mother's abdomen [36]. In 1833, a textbook on *Obstetric Auscultation* mentioned the possible correlation between FHR patterns and fetal health [37]. In 1906, Cremer first measured the FECG by using abdominal electrodes [38]. Since Cremer, FHR monitoring has been used clinically for assessing fetal health and status. Fetal cardiograms can predict fetal distress allowing doctors to prevent irreversible harm to the fetus. Over the next 50 years, varieties of improvements to FECG were made in the way of amplification and abdominal electrode placement, mostly in an attempt to improve resolution of the fetal QRS complex and calculate the FHR. Often, such efforts resulted in the complete obliteration of the P and T waves [39]. In the mid-1950s, the introduction of intrauterine electrodes (i.e., electrodes placed on the scalp of the baby via the birth canal during labor) and improved filtering techniques allowed physicians to obtain P and T waveforms whose shapes and positions they could relate to various fetal characteristics such as oxygen saturation and bradycardia [39]. Subsequently, many of the advances in improving the signal quality of the FECG focused on signals acquired directly from the fetus during the birthing process.

## 3. Interfaces and Noises Affecting the FECG Signal

The FECG exhibits a bandwidth of 0.05–100 Hz. In an abdominal register, the maximum amplitude of the QRS usually oscillates from 100 to 150 μV for the maternal recording and up to 60 μV for the fetal recording. The energy of the latter has been estimated to be less than one quarter of the total signal energy [40,41]. The FECG signals are often obscured by electrical noise from other sources. Common ECG noise sources, such as power line interference, muscle contractions, respiration, skin resistance interference, and instrumental noise, in addition to electromyogram and electrohysterogram due to uterine contractions, can corrupt FECG signals significantly [42]. The shape and structure of the FECG signal also depend on the placement of the electrodes (there is no standard electrode positioning for optimal FECG acquisition [43]), the gestational age, and the position of the fetus [44]. All of the aforementioned constraints make the FECG detection and extraction a difficult process. Therefore, it is important to understand the characteristics of the electrical noise. Electrical noise, which will affect FECG signals, can be categorized into the following types:

1. *MECG signal*: MECG is the most predominant interfering signal with FECG in the abdominal signal. The frequency spectrum of this noise source partially overlaps that of the ECG and therefore filtering alone is not sufficient to achieve adequate noise reduction.

2. *Power line interference*: power line interference consists of 60-Hz pickup and harmonics, which can be modeled as sinusoids and combination of sinusoids. Characteristics that might need to be varied in a model of power line noise include the amplitude and frequency content of the signal. These characteristics are generally consistent for a given measurement situation and once set, will not change during a detector evaluation. Due to the power line noise, the peak-to-peak amplitude caused by the frequency can reach up to 50% of peak-to-peak ECG amplitude.

3. *Maternal muscle noise*: muscle noise is due to maternal movement, often from the leg and abdominal muscles and may be picked up from the reference pad on the maternal thigh. Electromyographic activity in the muscles of the abdomen and uterus is the source of this kind of noise. Sometimes, it is difficult to identify the EMG signal in the abdominal signal.

4. *Electrode contact noise*: electrode contact noise is transient interference caused by loss of contact between the electrode and skin, which effectively disconnects the measurement system from the subject. Electrode contact noise can be modeled as a randomly occurring rapid baseline transition, which decays exponentially to the baseline value and has a superimposed 60-Hz component. The transition may occur only once or may rapidly occur several times in succession.

5. *Motion artifact*: when motion artifact is introduced to the system, the information is skewed. Motion artifact causes irregularities in the data. There are two main sources for motion artifact, electrode interface and electrode cable. Motion artifact can be reduced by proper design of the electronics circuitry and setup.

6. *Inherent noise in electronics equipment*: all electronic equipments generate noise. This noise cannot be eliminated; using high-quality electronic components can only reduce it.

7. *Ambient noise:* electromagnetic radiation is the source of this kind of noise. The surfaces of the human bodies are constantly inundated with electric–magnetic radiation and it is virtually impossible to avoid exposure to ambient noise on the surface of earth.

8. *Baseline drift and ECG amplitude modulation with respiration*: the drift of the baseline with respiration can be represented as a sinusoidal component at the frequency of respiration added to the ECG signal. The amplitude and frequency of the sinusoidal component should be variables. The amplitude of the ECG signal also varies by about 15% with respiration.

To end with, the signal-processing algorithm needs to remove the MECG complexes, reduce the effects of motion artifact and muscle noise, and then enhance the FECG to analyze the FECG for the monitoring of FHR.

## 4. FECG Signal Detection

It is a well-known fact that an FECG signal is obtained from the AECG of a pregnant woman that has the potential of being an effective diagnostic tool for determining the overall condition of the fetus during the delivery, as well as for the detection of pathological phenomena [45,46]. The fetal contribution to the AECG is very minor; therefore, it is not uncommon to record a much corrupt signal from which even the FHR can hardly be monitored. The detection of the FECG is yet a difficult task even when the maternal component of the signal has been reduced. In order to observe the FECG, some technique should be applied for improving the SNR and eliminating the maternal contribution to the signal [47-50].

Several methods have been proposed for detecting and processing the FECG signal from AECG signal. The first requirement for performing an untriggered averaging of the FECG is to determine the average FHR, later to be used as the averaging frequency. To detect FHR, two fundamental methods can be considered: a peak detection method and a transform method. Using the peak detection method, a small segment of the FECG is observed at a time and searched for the fetal R wave. Mainly, the result of the search depends on the algorithm used and on the local SNR in the above-mentioned data segment. Due to the unpredictable nature of the AECG signal, the local SNR value fluctuates about the SNR value of the entire signal and might sometimes be much smaller. Therefore, missing some peaks is a common experience while applying peak detection methods to noisy FECG signals. On the other hand, the use of the transform method, a new function of one or more parameters, is constructed from the historical signal. Each value of the new function represents a property of the entire signal. Consequently, each value depends not on the local SNR but on the SNR of the entire signal. Therefore, when the FECG is obscured by noise with unwanted signal and the peak detection algorithm fails to detect, a transform method might still detect the FHR. The regular transform method for identifying the periodicity of a hidden periodic signal within a time series is the Fourier transform. However, Fourier transform might sometimes fail to detect the average periodicity in the case of weak signals having small duty cycle. The main reason is the small correlation between the signal concerned and the analyzing functions (sine and cosine) of the Fourier transform.

In 1990, Y. Tal and S. Akselrod proposed a discrete Fourier transform method for the detection of FHR from AECG recordings [32]. The primary application of the proposed method is to simulate FECG signal. The proposed transform method empowers the detection of FHR from AECG signals where the fetal signal is barely visible. Following the elimination of the MECG contribution to the AECG signal, they computed a triple parametric transform function by multiplying the signal by their analyzing functions and integrating the result. In general, the method can be applied to handle weak, quasiperiodic, sharp signals of various origins. John W. Stoughton et al. [51] had a theory saying that adaptive least mean square linear prediction methods can be used for fetal heart tone signature analysis and detection in the presence of background acoustic noise. Adaptive signal-processing methods are presented in support of a noninvasive ambulatory FHR monitor. Successive evaluation of the detected fetal heart tone events are used to determine the instantaneous FHR. The initial investigation has indicated that linear prediction method is feasible for detecting the fetal heart tones in an advanced acoustic FHR monitoring system.

Kam and Cohen [28] proposed two architectures for the detection of FECG. The first architecture is a combination of an IIR adaptive filter and genetic algorithm (GA), where the GA is recruited whenever the adaptive filter is suspected of reaching local minima. The second one is an independent GA search without the adaptive filter. The main disadvantage of an IIR filter is that the error surface is not quadratic but a multimodal surface. Therefore, the presence of the GA forces the algorithm to overcome the local minima and reach the global solution. The quality of the extracted FECG using this IIR–GA adaptive filter is superior to that obtained using the GA alone. When there are uterine contractions in the ECG data, the method of combining an adaptive filter with a GA performs effectively.

In 2000, Kuei-Chiang Lai and John J. Shynk proposed an adaptive algorithm for detecting and separating fetal and maternal heartbeats from data containing both fetal and maternal QRS complexes that is capable of estimating the FHR and MHR from a composite ECG signal generated by the Genesis Technologies intrauterine catheter electrode [52]. This algorithm has comparatively low computational complexity and does not require reference signals to cancel the maternal QRS complexes and classifies the combined heart rate data as a series of fetal, maternal, and noise events using a technique of template matching. Chris Peters et al. [53] developed an algorithm that calculates the heart rate based on cross-correlation. By using this developed algorithm, the multielectrode electrical measurements on the maternal abdomen can be used for fetal monitoring in relatively early stages of pregnancy or other situations, where ECG amplitudes are low or noise levels are high. Therefore, in order to improve the detection accuracy for FECG signal, efficient signal-processing techniques are needed.

## 5. FECG Signal Processing for Extraction and Separation

The extraction of FECG is very important from a clinical point of view to get reliable information on fetal status, to detect abnormalities, to enable the adoption of measure for assuring fetal well-being, to check whether the fetus is alive or dead, and to determine twin pregnancies [54]. The method of recording the FECG from the mother's body, without direct contact with the fetus (which is highly desirable) is called noninvasive method. However, in this method of recording, the FECG signals have a very low power relative to that of the MECG. The method of recording FECG signal is far worse during the uterine contractions of the mother. During these contractions, the AECG recordings will be corrupted by other electrophysiological signals called uterine electromyogram or electrohysterogram, which are due to the uterine muscle rather than due to the heart. The response of the fetal heart to the uterine contractions is an important indicator of the fetal health. However, monitoring the FECG during these contractions is a complicated task because of very poor SNR. The three main characteristics that need to be obtained from the FECG extraction for useful diagnosis of the fetal condition include:

• FHR

• Amplitude of the different waves

• Duration of the waves

However, because of the noninvasive nature of measurement of the FECG, most of the signal-processing algorithms detect only the R waves and the P and T waves will usually remain hidden. In addition, FECG extraction problem is not easily solved by conventional filtering techniques. Linear filtering in the Fourier domain fails since the spectral content of all the three components, MECG, FECG, and noise, are rather similar and overlap. Some algorithm can be discussed followed by the past research that has been used in this area of fetal extraction.

### 5.1. Wavelet Transform

The WT is an efficient mathematical tool for local analysis of nonstationary and fast transient signals. One of the main properties of WT is that it can be implemented by means of a discrete time filter bank. The Fourier transforms of the wavelets are referred as WT filters. The WT represents a very suitable method for the classification of FECG signals from the abdominal ECG signal.

**Figure 2:**
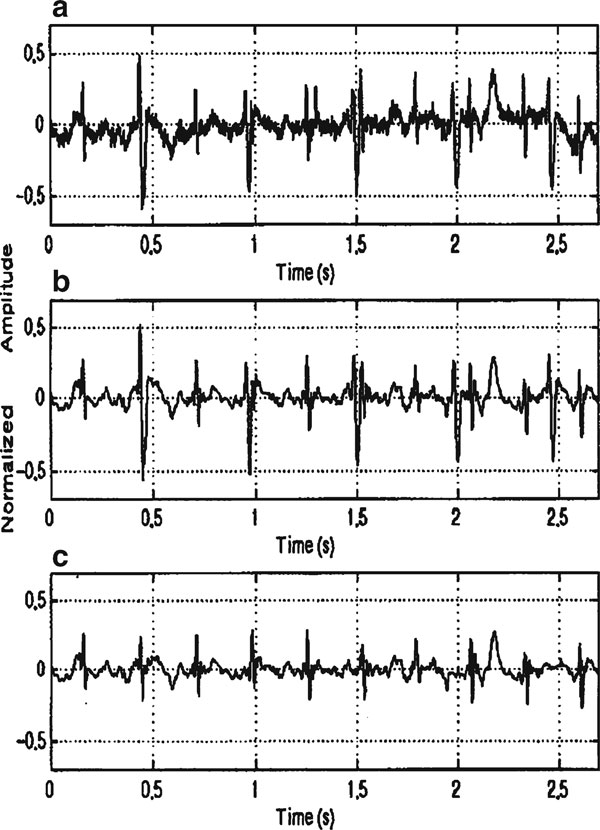
**a Abdominal ECG; b signal after the wavelet analysis stage; c extracted fetal QRS **[55].

In 1996, J.C. Echeverria et al. developed a procedure, wavelet analysis and pattern matching (WA–PM) for the off-line processing of AECG, on which they assumed that the signal (*s*(*t*)) can be mathematically described by the equation *s*(*t*) = *r*(*t*)·[*f*(*t*) + *m*(*t*) + *n*(*t*)] [55]. The terms *f*(*t*)*, m*(*t*), and *n*(*t*) denote, respectively, fetal, maternal, and Gaussian noise components, all of them affected by a modulation factor *r*(*t*) that causes baseline wandering [56]. Mainly, this developed procedure involves two stages: the first consists in a preprocessing stage for the suppression of low- and high-frequency additive noise based on an optimal wavelet multiresolution decomposition and the second cancels the maternal QRS complexes by means of pattern matching and template subtraction. The fetal QRS complexes can be easily identified from the resulting signal by applying a QRS detection algorithm. In order to eliminate detail signals [57] that do not have maternal and fetal QRS frequency components [58] and to allow maternal and fetal complex homogenization, the wavelet multiresolution decomposition was used. Mainly, the homogenization and noise elimination process based on wavelet multiresolution decomposition assure that the maternal QRS complexes on a real signal present morphological patterns than can be highly associated with the additive influence of the embedded fetal QRS complexes, while the pattern-matching procedure has the advantage of being specific to every record, giving more robustness to the identification process. A recorded AECG signal has been shown in Figure 2a. The stage of the method based on the wavelet analysis which produced the signal shown in Figure 2b, c contains the signal with the extracted fetal QRS after the pattern-matching and subtraction stage.

Ye Datian and Ouyang Xuemei [59] had a theory saying that wavelet analysis method can be utilized in the detection of FECG signal from the AECG signal. The wavelet analysis method is initially applied to detect the appearances of the distorted MECG signal and consequently MECG component is eliminated from the AECG signal. In fact, in some situation even after elimination of MECG, FECG is still challenging to be observed because the wavelet analysis can enhance only FECG under an appropriate scale factor of wavelet base function; therefore, it can more effectively and correctly detect FECG signal. The wavelet analysis approach can detect singularity of signal either in the frequency domain or in the time domain.

The FECG was monitored by calculating the Lipschitz exponent [60]. The main problem with this method is its inability to locate the FECG if it is obscured by the MECG. Since this happens two or three times in a 10-s period, it can be a major drawback. As the noise content is more during the uterine contractions, therefore there is a need to set the thresholds on the wavelet coefficients dynamically during denoising process. Again, the performance of this type of denoising during the contractions may not be optimum since thresholding of the wavelet coefficients may result in removing the FECG component altogether in the original signal.

Again, in 1996, S. Papadimitriou et al. [61] used wavelet transform to denoise FHR signals. His WT approach effectively removes transient spikes and reduces noise (both Gaussian and colored) without destroying the high-frequency information content of the signal (as the traditional low-pass filtering does). A noise reduction technique that detects noise components by analyzing the evolution of the WT modulus maxima across scales is adapted to improve the quality of FHR recording. The denoising scheme relies on the elimination of those multiscale maxima that correspond to noise components. The denoised FHR signal is reconstructed from the processed maxima by the inverse WT.

F. Mochimaru and Y. Fujimoto [62] also used wavelet-based methods to detect the FECG. They used multiresolution analysis (MRA) to remove the large baseline fluctuations in the signal as well as to remove the noise. MRA was performed on the raw ECG data with 12 levels of decomposition using wavelet function Daubechies20. Noise removal was accomplished by thresholding the wavelet coefficients at each level. The weighted standard deviation of the wavelet coefficients at each resolution level was used by thresholding for each resolution level. In 2004, E. C. Karvounis et al. [63] discovered that the complex continuous wavelet transform (CCWT) and modulus maxima theory can also be used to detect the QRS complexes of the fetal cardiac activity using multichannel MECG recordings. For a nonstationary signal, CCWT can be used to identify stationary sections of the data stream and locate and characterize singularities. Y. Song et al. [64] come with a similar kind of proposal where fetal heart sound signals can be detected, denoised, and reconstructed by utilizing wavelet-transform-based signal-processing approach. This approach improves the signal-to-noise ratio, which allows reliable FHR variation to be estimated under very weak signal environment.

Attempts to gain quantitative information of FECG from AECG recordings have been extensively investigated when signal is represented as function of time (time domain). Wigner-Ville distribution is one of the time–frequency approaches used for AECG signal processing to get the precise FECG signal for monitoring the FHR of the fetus. In 2006, E. C. Karvounis et al. [65] showed three-stage method for FHR extraction based on time–frequency analysis from AECG recordings. The method is based on the analysis of leads of the AECG signal. In the first stage, the maternal R peaks and fiducial points (QRS onset and offset) are detected, using time–frequency analysis, and the maternal QRS (MQRS) complexes are eliminated. The second stage locates the positions of the candidate fetal R peaks, using complex wavelets and pattern-matching theory techniques. The detection of the overlapped fetal R peaks with the MQRSs is accomplished in the third stage, using a histogram-based technique.

### 5.2. Artificial Intelligence

Some AI techniques mainly based on neural networks have been proposed for processing FECG signal. Neural network is a computing technique that evolved from mathematical models of neurons and systems of neurons. During recent years, neural networks have become a useful tool for categorization of multivariate data. This kind of technique is very useful for real-time application like FECG signal recording and analysis.

Digitized data from CTG measurements (FHR and uterine contractions) have been used for categorization of typical heart rate patterns before and during delivery. In 1994, John Liszka-Hackzell [66] showed that the categorization process for the FHR patterns, the backpropagation network, and the SOM network were used that can be reliable and agree well with the manual categorization. J. P. Marques de Sa et al. [67] proposed a method regarding FHR baseline determination using ANNs. Two baseline determination methods with multilayer perception ANNs (namely baseline estimation and baseline classification) were described and compared based on their practical application results. According to his proposed method, the results obtained by the estimation and classification approaches can be done based on quantitative indicators shown in Table 1.

**Table 1:** Comparison between estimation and classification analysis [67]

	Training set mean error	Test set mean error	Training set correct	Test set correct
Estimation	1.77	2.80	83 (97.6%)	50 (83.3%)
Classification	1.60	3.57	83 (97.6%)	49 (81.6%)

In the comparison between the results obtained by the estimation and classification approaches, in the classification analysis approach, the training set mean error (corresponding to the minimum test error) is slightly inferior to the estimation mean error. However, for the test set, the classification mean error is clearly superior to the corresponding estimation mean error (3.57 against 2.80 beats per minute).

In 1999, Giovanni Magenes et al. [68] proposed neural and fuzzy classifiers to discriminate among normal and pathological fetal states. Basically, both classifiers are based on linear and nonlinear indexes extracted from cardiotocographic fetal monitoring. They showed that the neural and fuzzy classifiers could improve the diagnostic information contained in CTG signals. S. Selvan and R. Srinivasan [27] had a theory saying that the two popular adaptive filtering techniques, namely adaptive noise cancellation and adaptive signal enhancement, are efficient techniques for processing of abdominal FECG by using neural network. Real-time recurrent learning algorithm is applied for training the proposed neural network that converges faster to a lower mean squared error and suitable for real-time processing as well. The proposed technique performs better than the noise canceller alone or a cascade connection of both noise canceller and signal enhancer.

**Figure 3:**
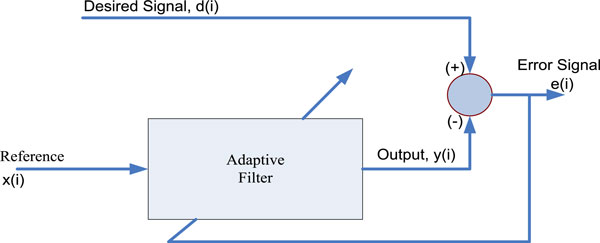
**Typical structure of an adaptive noise canceller **[25].

According to the method proposed by G. Camps et al. [25] in 2001, FECG can be extracted by using FIR neural network. FIR neural network is included in the familiar adaptive noise-canceling structure, shown in Figure 3, in order to provide highly nonlinear dynamic capabilities to the FECG extraction model. This network has solved complex situations more reliably than classical adaptive methods. In Figure 3, the reference input is considered as a thoracic maternal signal which is free from fetal contributions, where the desired signal is considered as the AECG signal. Although the original scheme of Widrow et al. [69] considered several reference signals, only one thoracic reference is considered in their proposed method. In this way, all the correlated components (maternal signal) vanish and the FECG register is obtained as the error signal.

FECG extraction using adaptive liner neural network was proposed by Mamun Bin Ibne Reaz and Lee Sze Wei [23] in 2004. The adaptive linear neural network filter is trained to cancel out the maternal signal to get only the fetal signal. As the fetal signal is weak under the domination of maternal signal and other noises and also the network emulates maternal signal as closely as possible to abdominal signal, thus only the MECG is predicted in the AECG. The main concept of this proposed method is that the network error equals AECG minus MECG, which is the FECG. This method is better than conventional filtering because subtraction is used instead. It can avoid eliminating desirable signal. Currently, in 2005, Philip Warrick et al. [70] used the combined tools of signal processing and neural networks to develop the automated technique to detect the FHR patterns of baseline, acceleration, and deceleration.

### 5.3. ICA and Blind Source Separation

Some others methods like ICA and BSS are becoming very popular for processing FECG signal from the AECG.

In 2000, Lieven De Lathauwer et al. [71] proposed the emerging technique of ICA, as an innovating way to solve a classical problem in biomedical engineering, namely the extraction of the FECG from multilead potential recordings on the mother's skin. The technique was illustrated by means of a real-life example. From a conceptual point of view, ICA is a very ambitious approach: it aims at the direct reconstruction of the different statistically independent bioelectric source signals, as well as the characteristics of their propagation to the electrodes, each revealing important medical information. It is nonparametric and is not based on pattern averaging, which could hamper the detection and analysis of typical fetal heartbeats. Barros and Cichocki [72] discovered a semi-blind source separation algorithm to solve the FECG extraction problem. This algorithm requires a priori information about the autocorrelation function of the primary sources, to extract the desired signal (FECG). They did not assume the sources to be statistically independent but they assumed that the sources have a temporal structure and have different autocorrelation functions. The main problem with this method is that if there is FHR variability, a priori estimate of the autocorrelation function of the FECG may not be appropriate for FHR analysis.

D. E. Marossero et al. [73] had a theory saying that ICA can be an efficient method for extracting the FECG from the composite electrocardiogram signals. They demonstrated the performance of an information theoretic ICA named Minimum Renyi's Mutual Information (Mermaid) [74] and the performance of the Mermaid algorithm, which is based on minimizing Renyi's mutual information, was evaluated. The effectiveness and data efficiency of Mermaid and its superiority over alternative information theoretic BSS algorithms are illustrated using artificially mixed ECG signals as well as FHR estimates in real ECG mixtures. In 2003, Ping Gao et al. [75] employed a combined method of singular value decomposition (SVD) and ICA for the separation of FECG from the mixture of ECG signals measured on the abdomen of the mother. They mainly applied a blind source separation method using an SVD of the spectrogram, followed by an iterative application of ICA on both the spectral and a temporal representation of the ECG signals. The SVD contributes to the separability of each component and the ICA contributes to the independence of the two components from the mixtures. In 2003, Vigneron et al. [76] also applied BSS methods for FECG extraction. They showed that the FECG could be reconstructed by means of higher-order statistical tools exploiting ECG nonstationarity associated with postdenoising wavelets.

In 2004, F. Vrins et al. [77] applied the method BSS for FECG extraction. In this application, the sources are the FECG and MECG, diaphragm, and uterus and the mixtures are recorded through electrodes located on the pregnant woman's abdomen. By using ICA method, in 2004, M. Burghoff and P. Van Leeuwen [78] have given their theory about the separation of fetal and maternal magnetocardiographic signals in twin pregnancy. ICA, which uses higher-order statistics to decompose the signal into statistical-independent components, has already been used in single pregnancies to distinguish between maternal and FECG signals [29,76]. By using this method, the results showed that the maternal and fetal components could be separated from each other as well as from other sources of noise and artifacts in the abdominal signal. In 2004, Charuyuphan Chareonsak et al. [79] proposed a real-time BSS method that can be used to separate the FECG from the MECG effectively.

Recently, Farshid Soheili Najafabadi et al. [12] also applied the ICA for the separation of FECG and MECG signal from the AECG in 2005. According to their result, it is concluded that ICA works magnificently in order to extract FECG from the AECG even in SNR = -200 dB using simulated data without quantification noise. It showed that the performance was drastically decreased in existence of quantification noise. In 2005, a new FECG extraction algorithm for a single-channel wearable FHR monitoring system has been proposed by J. Lee et al. [80]. This algorithm is considered into a training and detection step. In a training step, a demixing vector was computed with overdetermined BSS and fetal beat detection was performed by utilizing the computed demixing vector in the detection step. The algorithm was evaluated with a simulation signal that has diverse heart rates and with real maternal AECGs. In all the cases, detection was perfectly achieved and excellent immunity to 60-Hz noise was found. FHR variability was calculated with FECG beats detected using the algorithm. Besides, the shapes of FECGs could be observed with the representative template.

### 5.4. Other Methods

A range of signal-processing methods is applied by various researchers for the purpose of accurate and actual FECG signal for the monitoring of FHR. Some of these models are briefly explained here. The problem of FECG extraction was tackled more than 30 years ago by means of new conventional adaptive noise-canceling techniques. In 1985, Widrow and Stearns [81] used a linear adaptive filter framework to cancel the mother ECG and obtain the FECG. By using the filter framework, they used two sets of electrodes, one set placed on the abdomen of the mother and the other placed on the chest of the mother. The electrodes placed on the abdomen pick up both the FECG and MECG (considered as primary inputs), whereas the electrodes placed on the chest pick up only the MECG (considered as reference inputs). According to the method in which the signals from the electrodes placed on the abdomen as desired and the signals from the electrodes placed on the limbs as input to the adaptive filter, the error signal can be obtained to represent the extracted FECG. Although this method is providing a solution, this is not robust enough to be used for clinical practice. Firstly, the problem is that one needs to have more electrodes to measure the ECG signals. Secondly, if the amplitude of the background noise is greater than the fetal heartbeat during the uterine contractions, the resulting error signal will not contain the FECG accurately. This method fails to extract the FECG when both the MECG and FECG signals are overlapped. Furthermore, if there is escape of the FECG into the recordings of the reference input (MECG), the quality of the extracted FECG will be extremely poor.

The genetic algorithm approach for FECG extraction, proposed by Horner et al. [82], is based on subtracting a pure MECG from an abdominal signal containing FECG and MECG signals. Subtraction via a genetic algorithm is supposed to be near optimal rather than a straight subtraction. The issue with this proposed approach is needed to get the MECG signals whose shape is similar to the MECG present in the abdominal recordings where the FECG signals also available. Therefore, it needs to be determined exactly where the electrodes need to be placed to pick up the MECG alone. F. Magalhaes et al. [83] have used approximate entropy (ApEn) and wavelet filtering method for characterization of FHR irregularity for fetal risk assessment. ApEn was able to discriminate three categories of behavioral patterns: calm sleep, calm vigilance, and pathological flat-sinusoidal condition. They showed high level of discrimination between normal and pathological intrapartum FHR tracings by "exact" removal of the accelerative and decelerative components.

In 2004, George G. Georgoulas et al. [84] presented an approach to automatic classification of FHR tracings belonging to hypoxic and normal newborns. The classification was performed using a set of parameters extracted from the FHR signal and two hidden Markov models. Their results were satisfactory indicating that the FHR convey much more information than what method is conventionally used for FHR classification. In 2003, G. Vasios et al. used the matching pursuits (MP) method in order to extract the very-low-frequency periodic components of the complicated FHR fluctuations during labor and to examine the long-term modulation characteristics of the heart rate in relation to the oxygen saturation of fetal arterial blood [85]. They focused on the very low frequency range since some of the adaptive responses of the fetus are associated with the long-term slowly varying components of the FHR. MP method is sufficiently sensitive to detect abrupt perturbations and multiple periodicities in the dynamic pattern of the intrapartum and antepartum FHR.

Partha Pratim Kanjilal et al. [13] presented SVD method for the FECG extraction from single-channel MECG in 1997. The proposed method employs singular value decomposition and analysis based on the singular value ratio spectrum. The MECG and the FECG components are identified in terms of the SV-decomposed modes of the appropriately configured data matrices and the elimination of MECG and determination of FECG were achieved through selective separation of the SV-decomposed components. The distinctive feature of this method is that only one composite MECG signal is required to determine the FECG component. Therefore, the method is numerically robust and computationally efficient.

**Figure 4:**
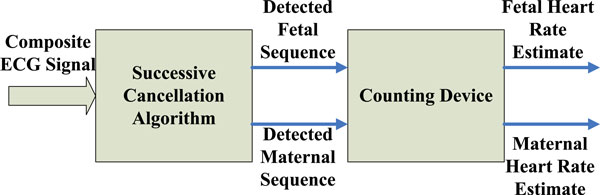
**Fetal and maternal heart rate estimation using the SC algorithm **[86].

In 2002, Kuei-Chiang Lai and John J. Shynk used a successive cancellation algorithm for FHR estimation using an intrauterine ECG signal [86]. Mainly, they used a two-stage successive cancellation (SC) algorithm that sequentially separates fetal and maternal heartbeats from an intrauterine electrocardiogram signal containing both fetal and maternal QRS complexes. The heartbeats are separated consequently in two stages: each stage initializes a template for fetal or maternal source and then performs event classification based on a template-matching technique. In the initialization period, the first stage initializes, without priori knowledge of signal power ranking, the template of the stronger source, which is canceled from the composite ECG signal prior to initialization of the weaker source's template in the second stage. Similarly, beyond the initialization period, the classified events of the stronger source are removed before classification of the weaker ones. The postprocessing step improves the classification results by searching for heartbeats that are not detected due to overlapping fetal and maternal complexes or noise corruption. The outputs of the postprocessing blocks are the locations of the detected fetal/maternal complexes. A simple counting device that measures the intervals between adjacent detected fetal/maternal events is then used to derive the instantaneous fetal/maternal heart rates, as illustrated in Figure 4.

In 2005, C. Kezi Selva Vijilal et al. [87] proposed an adaptive neurofuzzy logic technique for the extraction of FECG signal by canceling the MECG signal from the AECG signal. Adaptive noise cancellation was used to remove background noise from useful signals. This is an enormously useful technique where a signal is submerged in a very noisy environment. Usually, the background noise does not keep steady and it will change from time to time. Therefore, the noise cancellation must be an adaptive process: it should be able to work under changing conditions and be able to adjust itself according to the changing environment. The basic idea of an adaptive noise cancellation algorithm is to pass the corrupted signal through a filter that tends to suppress the noise while leaving the signal unchanged. In addition, as mentioned above, this is an adaptive process, which means it cannot require a priori knowledge of signal or noise characteristics.

FECG extraction problem from the AECG is not easily solved by conventional filtering techniques. Linear filtering in the Fourier domain essentially fails since the differences among the three components in the AECG signal: maternal, fetal, and noise cannot be defined in the spectral domain. In addition, the spectral content of the three components is rather similar in the shape and amplitude and contains strong broadband contributions. Several different approaches (methods) like coherent averaging, matched filtering, autocorrelation- and cross-correlation-based methods, adaptive filtering, sequenced adaptive filtering, singular value decomposition, multireference adaptive noise cancellation, etc. have been proposed to address this problem [16]. All these methods have one of the following limitations: (1) a single channel of FECG is extracted whereas better characterization can be accomplished by two or more channels; (2) the signal acquisition from the abdomen is highly sensitive to electrode placement, human interaction, stage of pregnancy, position of fetus, etc.; (3) extraction of P and T waves is not satisfactory [88-90].

In 1995, Mooney et al. [91] designed a microcomputer-controlled data acquisition system capable of accurately capturing the fetal cardiac electrical signal in maternal transabdominal recordings. Table 2 presents several methods proposed in the literature for the extraction of the FHR. All the mentioned methods in Table 2 were validated using real recordings (no simulated signals were involved) while all leads are placed on the abdomen of the mother to get the abdominal signal (no thoracic leads were used). Due to the fact that there is no benchmark database for this area and therefore, each approach is evaluated and performed using different datasets. The method proposed by Mooney et al. [91] is not fully automated; areas of the AECG are initially selected from a user and then the FHR is calculated where five to eight leads were used for this proposed method. The fuzzy-based approach for the extraction of FHR by Azad [92] performed very well with average performance, but there is no reference about the accurate number and duration of AECG records used for the evaluation. Pieri et al. [93] use the larger dataset among all the methods presented in Table 2 (400 records of 5–10 min each), but the results are rather poor than the other methods mentioned in the Table 2. The study by Ibrahimy et al. [94] also performs very well and it is validated using a large dataset (five records of 20 min each), but the reported result is the correlation coefficient between the simultaneous FHR measured from Doppler ultrasound.

**Table 2:** Compassion several existing FHR extraction method

Author	Description	Dataset	Accuracy (%)
Karvounis et al. [63]	Complex wavelet	15 records (three abdominal leads); duration 1 min	98.94
Mooney et al. [91]	Adaptive algorithm	Several records (five abdominal leads)	85
Azad [92]	Fuzzy approach	Five records (three abdominal leads)	89
Pieri et al. [93]	Matched filter	400 records (three abdominal leads); duration 5–10 min	65
Ibrahimy et al. [94]	Statistical analysis	Five records (one abdominal leads)	89

## 6. Hardware Models

Because of the advanced enlargement of the biomedical discipline, the application of biomedical instruments becomes indispensable in daily life. Design of application-specific integrated circuit for the biomedical instrument has become quite important recently. Various hardware has been implemented to develop FHR monitoring to assess the fetal state thus assuring his well-being during pregnancy period.

**Figure 5:**
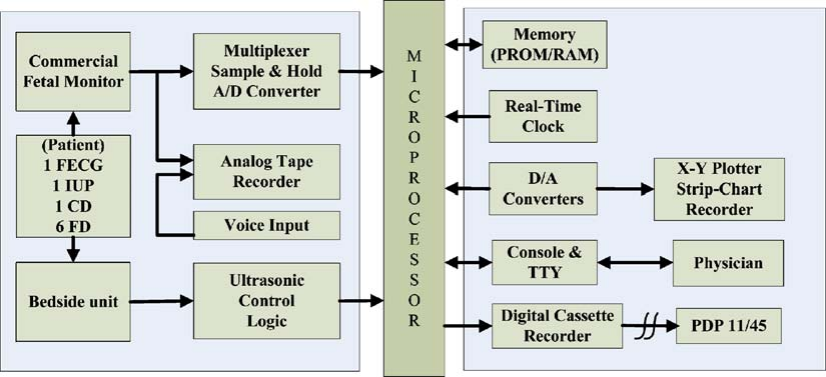
**The functional block diagram of the microprocessor-based data acquisition system **[95].

The design, development, and preliminary evaluation of a microprocessor-based data acquisition and processing system were presented by Wen C. Len et al. [95] in 1977, which continuously and simultaneously acquires signal for recording and evaluating cervical dilation and fetal descent as well as FHR and maternal intrauterine pressure in an attempt to gain a better understanding of the kinematics and dynamics of labor. Figure 5 shows the functional block diagram of the microprocessor-based data acquisition system for FHR monitoring system.

**Figure 6:**
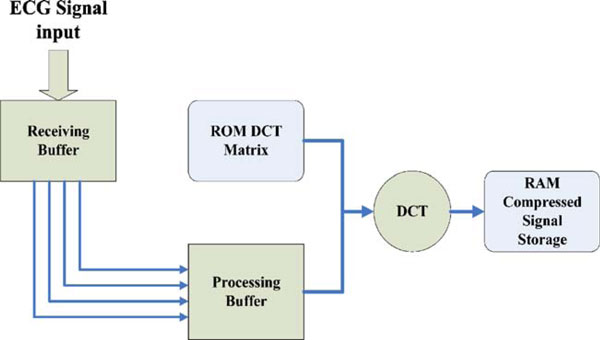
**Overall system architecture **[96].

In 2001, B. S. Pimentel et al. [96] offered a hardware implementation of a digital compression tool for electrocardiographic signals based on a discrete cosine transform (DCT). The platform chosen is a field programmable gate array (FPGA), due to its ease of use and rapid prototyping characteristics. As the main concern is to perform real-time compression, the device would need two entry buffers: one to save incoming data and another to perform the calculations as shown in Figure 6. Once the receiving buffer is full, the system copies its contents to the processing buffer and begins the DCT calculations. Obviously, these have to be finished before the receiving buffer is full again. To keep the design less complex, they have made the simplifying assumption that their processing buffer (initial RAM module) always has the most recent data waiting to be compressed.

The DCT component matrix is stored in a ROM module within the FPGA logical units. The ECG frame is copied from the receiving buffer and stored in a RAM module and both values are loaded into the input registers of a multiplier. This is the same as multiplying the correspondent elements of the DCT matrix and ECG vector. The results of the multiplication of each row of the DCT matrix and the ECG vector are then added in an accumulator. Finally, as soon as an entire row has been used, the result is stored in another RAM module, which keeps the transformed signal.

Recently, in 2005, Charayaphan Charoensak and Farook Sattar [97] designed an efficient FPGA hardware architecture for the realization of a real-time BSS. The architecture can be implemented using a low-cost FPGA. The architecture offers a good balance between hardware requirement (gate count and minimal clock speed) and separation performance. The FPGA design implements the modified Torkkola's BSS algorithm for audio signals based on ICA technique.

## 7. Discussion

By the review, it can be realized that, for the detection of FECG from the composite AECG signal, the Fourier transform method plays a significant role. Using the transform method, a new function of one or more parameters is constructed from the temporal signal where each value of the new function represents a property of the entire signal. Consequently, each value depends not on the local SNR but on the SNR of the entire signal. Therefore, when the FECG is obscured by noise and the peak detection algorithm fails, a transform method might still detect the FHR proficiently. Apart from this, the adaptive least mean square linear prediction method is a prominent method to detect and analyze the FECG in the existence of surrounding noise interface. Similarly, the adaptive algorithm is a quite accurate method with relatively low computational complexity to detect the fetal and maternal signal from the AECG signal. The CCWT and modulus maxima theory can also be used to detect QRS complexes. This system performs well since almost all fetal bears are detected and accuracy is about 99.5%. By using wavelet analysis, it is utilized to enhance the FECG so that the coherent average can get more accurate reference and the elimination of baseline drift. Extraction of the FECG signal by the wavelet multiresolution decomposition and a pattern-matching procedure shows a high-precision method for fetal QRS extraction and maternal QRS cancellation. WT analysis offers a more flexible and effective tool for FHR signal denoising than the traditional filtering techniques and the denoised FHR signal is reconstructed from the processed maxima by the inverse WT. The time–frequency analysis is an alternative way to eliminate maternal QRS complex and the result by using this method shows good accuracy (96%). Neural network used for the categorization of FHR patterns is consistent and fast and does not involve human efforts. For FHR signal baseline estimation and classification, excellent results are obtained in the application of artificial neural networks, besides the obvious limitation of the training set size and constitution. Therefore, the estimation approach seems to be the best choice. It is also clear that result of neural and fuzzy classifiers shows very promising performance on the set of collected FHR signals. Again, adaptive noise cancellation and adaptive signal enhancement are efficient techniques for processing of abdominal FECG by using neural network which is faster to a lower mean squared error and suitable for real-time processing. FIR neural network is also a familiar adaptive noise-canceling producer in order to provide highly nonlinear dynamic capabilities to the FECG extraction model. ICA is a very ambitious approach for the extraction of the FECG in real-time environments. ICA works magnificently in order to extract FECG even in SNR = -200 dB using simulated data without quantification noise. ICA offers a fast and efficient approach for the preprocessing of MCGs with multiple signals of interest, in particular when the signal-to-noise ratio is low. However, the main disadvantage of the BSS-based approach is that it requires a large number of recorded ECG leads. In addition, this procedure is cumbersome as, for each signal component to be processed, it requires visual inspection of the data and the manual selection of an appropriate number of data segments for a representative template. An alternative approach in the identification of signal sources is ICA, which uses higher-order statistics to decompose the signal into statistical-independent components. Some combined method like SVD and ICA can be the way of solution of other BSS technique that works well for extracting an FECG from the composite signal. The sketch out of the foremost methods is given in Table 3.

**Table 3:** Sketch out of the foremost methods

Signal	Method	Advantage/disadvantage
Detection	Fourier transform	When the FECG is obscured by noise and the peak detection algorithm fails, a transform method might still detect the FHR proficiently
		SNR is averagely high
		In the case of weak signals having small duty cycle, this tool might sometimes fail to detect the average periodicity because of small correlation between the signals
	Least mean square	Feasible for fetal heart tone signature identification and analysis in the presence of background acoustic noise.
	Complex continuous wavelet transform (CCWT)	Performs well and the accuracy of the method is high
		Algorithm's parameters increase the system's efficacy
		Computationally fast and excels in performance
		Able to extract the MHR signal, which can be useful for parallel monitoring of the mother's health
Extraction	Wavelet transform (WT)	Coherent average can get more accurate reference
		Can be obtained to smooth the baseline drift
		Requires only one abdominal signal for fetal QRS extraction and maternal QRS cancellation
		More flexible and effective tool for FHR signals denoising than the traditional filtering techniques
	Time–frequency analysis	Three leads are used for FECG extraction
		Spectrum produced by Wigner-Ville distribution (WVD) distribution displays very good localization properties
		The main drawback of the method is the difficulty to extract the fetal R peaks in noisy background or in cases where the FECG is not distinguishable
	Artificial neural networks (ANN)	Very fast and does not involve human efforts for categorization
		Neural networks can offer the computational power of non-linear techniques
		Sometimes it does not estimate the exact baseline value and its precision is limited by the number of classes
	ICA and BSS	Relatively, SNR is high
		Efficient both in batch and on-line operation modes
		Fast and efficient approach for the preprocessing of multiple signals of interest
		No specific prior knowledge required in order to identify components generated from different sources
		Often require a large number of recorded leads to reach reliable FECG extraction

## 8. Conclusion

Detailed analysis of the FECG during labor could provide valuable additional information about the health conditions of the fetus as well as to assist clinicians in reducing incidents of unnecessary medical intervention. As a result, long-term FHR monitoring is important during the pregnancy and labor. Therefore, the aim of this paper was to provide concise information about FECG and reveal the different methodologies to analyze the signal for efficient FHR monitoring. Techniques for FECG signal detection and extraction from the composite AECG signal were discussed along with their advantages and drawbacks. Finding of a difficulty or problem in one method leads to other improved methods. This revision clearly points up the various types of FECG signal analysis techniques so that accurate methods can be applied during any medical diagnosis, biomedical research, hardware implementations, and end user applications.
